# The Neuroprotective and Biomarker Potential of PACAP in Human Traumatic Brain Injury

**DOI:** 10.3390/ijms21030827

**Published:** 2020-01-28

**Authors:** Denes Toth, Andrea Tamas, Dora Reglodi

**Affiliations:** 1 Department of Forensic Medicine, University of Pécs Medical School, Szigeti út 12, H-7624 Pécs, Hungary; denes.toth@aok.pte.hu; 2 Department of Anatomy, MTA-PTE PACAP Research Team, University of Pécs Medical School, Szigeti út 12, H-7624 Pécs, Hungary; andreatamassz@gmail.com

**Keywords:** PACAP, neuropeptide, traumatic brain injury, biomarker, neuroprotective

## Abstract

Traumatic brain injury remains a growing public health concern and represents the greatest contributor to death and disability globally among all trauma-related injuries. There are limited clinical data regarding biomarkers in the diagnosis and outcome prediction of TBI. The lack of real effective treatment for recovery calls for research of TBI to be shifted into the area of prevention, treatment of secondary brain injury and neurorehabilitation. The neuropeptide pituitary adenylate cyclase activating polypeptide (PACAP) has been reported to act as a hormone, a neuromodulator, a neurotransmitter and a trophic factor, and has been implicated in a variety of developmental and regenerative processes. The importance of PACAP in neuronal regeneration lies in the upregulation of endogenous PACAP and its receptors and the protective effect of exogenous PACAP after different central nervous system injury. The aim of this minireview is to summarize both the therapeutic and biomarker potential of the neuropeptide PACAP, as a novel possible target molecule presently being investigated in several human conditions including TBI, and with encouraging results in animal models of TBI.

## 1. Introduction

Traumatic brain injury (TBI) is caused by an external force [1] and is often referred to as the “silent epidemic” [2]. TBI remains an increasing public health concern and represents one of the most important contributors to death and disability among all trauma-related injuries [3]. An estimated 69 million people suffer TBI each year, with a severity of mainly mild (81%) and moderate (11%) [4]. Apart from the many physical and cognitive effects to deal with after a brain injury, there can also be many medico-legal (criminal, insurance, personal injury) issues to consider, like estimation of the survival time post-injury by histopathologic examination or prognostication the residual deficits. Biomarkers associated with different characteristics of TBI may also be of clinical value for a more precise classification and risk assessment of TBI, thus optimizing treatment options [5]. The heterogeneity of the primary insult (focal, multifocal or diffuse), along with the variable secondary biochemical and cellular responses, makes the management and prognostication of TBI difficult [6]. At present there are limited clinical data available regarding the use of biomarkers in both the diagnosis of TBI and outcome prediction after TBI. It is critical to distinguish between different TBI severities, however, it is not clear which biomarkers are best for diagnosis and prognosis in different severities of TBI [7]. Over the past few years, there has been a constant search for new biomarkers specific to TBI. Currently available results of TBI pathophysiology research suggest that there is a need to identify additional, new biomarkers for TBI that alone or together with others can reflect the diverse injury characteristics of TBI.

Another clinically challenging aspect of TBI is the poor outcome and limited therapeutic possibilities. Based on animal studies hundreds of candidates have emerged as potential treatment option to reduce the brain damage. However, only a few have real translational value. The aim of this review is to summarize both the therapeutic and biomarker potential of the neuropeptide PACAP (pituitary adenylate cyclase activating polypeptide), as a novel possible target molecule presently being investigated in several human conditions including TBI, and with encouraging results in animal models of TBI [8].

## 2. General Overview

PACAP is a neuropeptide that was first isolated in 1989 from ovine hypothalamic extract [9]. The sequence of PACAP has been well conserved during evolution, suggesting that PACAP is involved in the regulation of basic biological functions [10]. After its discovery, PACAP was reported to act as a neurohormone, a modulator, a transmitter and a neurotrophic factor, and has been shown to be involved in various developmental processes [11]. There are two isoforms of PACAP, PACAP-38 [9] and PACAP-27, resulted from proteolysis of the same precursor protein and they share the same 27-amino acid N-terminal bioactive core [12]. In mammalian tissues, PACAP-38 is the dominant form, representing 90% of the naturally occurring peptide. Therefore, most experiments are performed with this isoform and unless specifically indicated, PACAP usually refers to the longer isoform in the literature and this is what we are also following in our review. PACAP belongs to the secretin/glucagon/growth hormone-releasing hormone/vasoactive intestinal peptide superfamily. The effects of PACAP are mediated through class B-G protein-coupled receptors identified as PAC1, VPAC1, and VPAC2. PAC1, which exhibits a greater affinity for PACAP than for vasoactive intestinal peptide (VIP), is found in the central nervous system in abundance and is associated with neuroprotective and neurotrophic effects. VPAC1 and VPAC2 are more related to peripheral actions and are equally recognized by both PACAP and VIP [13]. PACAP plays a very important role in brain development and is widely expressed in the embryonic brain at the onset of neurogenesis [14,15]. After the termination of brain development, PACAP expression is reduced in most brain areas [16]. Reduced PACAP level, as several other brain trophic factors, has also been implicated in physiological and pathological aging processes [17]. Regeneration of the nervous system after injury is likely to reemploy mechanisms used to regulate brain development in the pre- and postnatal periods, with the upregulation of several growth factors, like nerve growth factor, insulin-like growth factor, and brain-derived neurotrophic factor [18,19]. Similarly, PACAP is also strongly upregulated in several models of neuronal injuries [11,20,21]. PACAP is now a well-known neuroprotective factor with strong effects in several in vitro and in vivo animal models [20,22,23,24,25]. Among others, PACAP has potent neuroprotective effects in models of focal and global cerebral ischemia [23,25,26], retinal pathologies [27], neuronal toxicities [22], multiple sclerosis and other inflammatory conditions [28,29], as well as in models of neurodegenerative diseases like Parkinson’s disease, Alzheimer’s disease and amyotrophic lateral sclerosis [30,31,32,33]. Few studies indicate the potential protective effect of PACAP also in traumatic central and peripheral nervous system injuries, which have been summarized in an earlier review paper [34]. In our present manuscript, we give an overview of these results concentrating on the traumatic brain injuries and add more recent data since the last review in this topic was written [34]. 

Regarding the biomarker value of PACAP, dozens of recent studies have investigated presence and changes of the neuropeptide in various human conditions. The presence of PACAP has been described in several human tissue samples and biological fluids [8]. Among tissues, PACAP occurs at highest concentrations in the brain and endocrine glands [8], but numerous peripheral tissues also have detectable levels. Altered PACAP expression in human tissues was demonstrated in several pathological conditions, like ductal adenocarcinoma of the pancreas [35], papillary carcinoma of the thyroid gland [36], ischemic heart disease [37] and inflammatory bowel disease [38]. In the brain, PACAP expression could be examined from postmortem human samples, where a manifest reduction was observed in Alzheimer’s disease, in the temporal, frontal and occipital lobes [39,40]. Biological fluid samples have great clinical importance for their diagnostic value. PACAP has been investigated with different methods, including mass spectrometry, radioimmunoassay (RIA) and enzyme-linked immunosorbent assay [8]. These studies have shown that, in addition to the human serum, PACAP occurs in the cerebrospinal fluid (CSF), ovarian follicular fluid [41,42], human milk [43,44] and synovial fluid [45]. In many conditions, changes in PACAP have been described to reflect disease progression, like the decreased synovial fluid PACAP levels in post-traumatic osteoarthritis [45] or to correlate with other physiological parameters, like the number of oocytes in the follicular fluid [42]. PACAP has a short half-life between 3 [46]– 10 min [47] in the serum, in spite of which, numerous research groups have reported that levels can be stably measured. Changes in serum PACAP levels have been described in nephrotic syndrome and in cardiomyopathies, where a decrease has been detected [48,49]. Regarding nervous system conditions, elevation has been found in acute aneurysmal subarachnoid hemorrhage [50], in acute spontaneous basal ganglia hemorrhage [51], in ictal phases of migraine [52,53,54], while decreases have been described in interictal phases of migraine [52,55] and in female post-traumatic stress syndrome patients [56]. CSF PACAP levels are decreased in Alzheimer’s disease [39,40] and multiple sclerosis [57]. These clinical data indicate that PACAP has a diagnostic and/or prognostic potential in different brain pathologies, like hemorrhages, multiple sclerosis and Alzheimer’s disease. PACAP levels have also been measured after traumatic brain injury in human patients in the brain tissues [58], CSF and serum [59]. Results of these studies are also briefly summarized in the present review.

## 3. Protective Effects of PACAP in Animal Models of TBI

The first proof of PACAP being protective in traumatic brain injury came from observations by Farkas et al. [60]. They examined a rat model where diffuse axonal injury was induced by impact acceleration (Marmarou model). After the induction of the brain injury, intracerebroventricular (icv.) treatment with 100 μg PACAP was started immediately. This intervention led to a significant reduction of beta-amyloid precursor protein-immunopositive axon profiles in the area of one of the most important central nervous system pathways, the corticospinal tract, compared to controls 2 h after the injury [60]. A follow-up study by Tamás et al. examined the possible neuroprotective effect of a delayed PACAP treatment after injury in the same rodent model. Icv. treatment with 100 μg PACAP 30 min or 1 hour after the induction of TBI resulted in a significant reduction in the density of beta-amyloid precursor protein-immunopositive axon profiles in the corticospinal tract [61]. Subsequent studies have confirmed these observations. Miyamoto et al. examined the neuroprotective effects of PACAP38 by regulating oxidative stress in mice with TBI. Reactive oxidative metabolites and biological antioxidant potential were measured before and 3, 4 and 24 h after controlled cortical impact (CCI). In this case, intravenous PACAP38 administration was also started immediately after CCI, and immunostaining for the nitrotyrosine as an indicator of neuronal death, was measured 24 h later. The ratio of biological antioxidant potential and reactive oxidative metabolites was used to estimate the balance between oxidative stress and endogenous antioxidant activity. This ratio increased significantly at 3 and 24 h post-CCI, which suggests that the level of oxidative stress was significantly upregulated in the CCI-treated animal. The total injury volume was calculated by integrating areas from the immunostained sections and it was found that PACAP38 treatment significantly reduced the TBI volume compared with vehicle-treated animals. In case of PACAP38 treatment, suppressed oxidative stress levels were seen 24 h post-CCI based the nitrotyrosine staining. The authors also clarified that PACAP treatment increased brain levels of two antioxidants, SOD-2 and GPx-1. This result suggested that PACAP38 administration immediately following CCI was able to prevent neurodegeneration and decrease levels of the oxidative stress indicator nitrotyrosine likely due to a concurrent increase in antioxidant capacity 24 h following injury [62]. 

Another model that is often used to mimic diffuse TBI is the central fluid percussion head injury model. In this rat model, Kövesdi et al. examined the axonoprotective effect of PACAP in the brainstem [63]. They administered 100 μg PACAP icv. 30 min after the injury. Two h after injury the animals were sacrificed for histological assessment. Beta-amyloid precursor protein, as a classical marker indicating impaired axoplasmic transport, and RMO-14 antibody, representing foci of cytoskeletal alterations were used. The results demonstrated that icv. administration of 100 µg PACAP 30 min after injury significantly reduced the density of damaged axons in the corticospinal tract after the injury, indicating that PACAP could be an efficient inhibitor of impaired axoplasmic transport and neurofilament compaction associated with axonal injury [63]. Mao et al. [64] examined the neuroprotective mechanisms of PACAP pretreatment in a modified Feeney weight drop contusion model in rats. A neurological behavioral score system was applied to assess the neurobehavioral, motor, and cognitive functional deficits induced by the traumatic injury. These included determination performances in the inclined plane task and Morris water maze. Sample collection for histological analysis was done after 1 and 21 days. Icv. pretreatment with PACAP markedly diminished the motor and cognitive dysfunction induced in the model. Attenuated apoptosis, inflammation (decreased interleukin-1β and tumor necrosis factor-α levels), and edema were observed, and also inhibited upregulation of TLR4 and its downstream signaling molecules MyD88, p-IκB, and NF-κB. These changes were observed in the area around the injured cortical parts and also in the hippocampal areas. [64]. The effects of intraventricular infusion of PACAP (1µL/5µL saline) on TBI induced T-cell mediated immune response were examined by Hua et al. in the Feeney weight drop contusion model in rats. PACAP infusion into the ventricle 20 min prior to induced TBI reduced the edema in the brain tissue and alleviated neuronal swelling and necrosis. In case of PACAP pretreatment flow cytometry showed increased CD4^+^T cells and decreased CD8^+^. The authors concluded that possibly PACAP inhibits the expression of IL-12 thereby preventing T cell proliferation, and PACAP inhibited FasL expression suppressing the apoptosis of CD4^+^T cells [65].

## 4. PACAP Levels in the Brain After TBI in Animal Models

Soon after the discovery of PACAP, it was shown by RIA measurements that PACAP occurs at highest concentrations in the brain [8]. Several studies have investigated the changes of endogenous PACAP expression after various insults, including rat TBI model by Skoglosa et al. [66]. The authors examined expression of mRNA for PACAP and PAC1 receptor after a moderate traumatic brain injury in the rat cerebral cortex and hippocampus. In this model they used a 21 g free-falling weight that was dropped from a height of 35 cm on a piston. The results showed that TBI in rat brain cortex lead to a prolonged increase in the expression levels of PACAP. Up-regulation of PACAP mRNA levels was observed both in the cortex and in the hippocampus. PACAP mRNA expression was decreased in the center, but strongly increased in the perifocal area of the cortex around the lesion. The level of PAC1 receptor mRNA was at a minimum 6 h post-injury and it reached the control level at 72 h after the injury. The authors concluded that the increased levels of PACAP mRNA after brain injury might be neuroprotective for vulnerable neurons and that PACAP had beneficial effects on neuronal survival [66]. In contrast, Jaworski et al. observed no changes in a cortical stab injury model in rats. PACAP or PAC1-R mRNA expression did not show upregulation as a result of the glial hypertrophy and hyperplasia accompanying the penetrating wound. There was no change in the expression of PACAP or PAC1 receptor mRNA in the lesion penumbra, callosal neurons in the contralateral cortex, or thalamic afferents either [67]. A possible explanation for this discrepancy can be the differences between the two trauma models. It is known that the sharp force (stab) injury does not induce an inflammatory response as severe as a blunt force (contusion). In another study using classical cortical impact injury, differences were found in the receptor expression. In this model Morikawa et al. [68] examined the expression and the cellular localization of PAC1R immunohistochemically after TBI. PAC1 receptor immunoreactions were detected in the perifocal area of the lesions from 3 h after TBI, and the intensity and number gradually increased up to 7 days. Using double-immunohistochemistry, the PAC1 receptor immunopositive cells were co-localized with the microglia on the first day and with microglia and astrocyte seven days after TBI [68]. In contrast, Suzuki et al. [69] revealed the localization of PAC1 receptor in a cortical stab wound model. PAC1-receptor-like immunoreactivity was observed in the reactive astrocytes at five days after a stab wound, whereas no PAC1-receptor-like immunoreactivity was detected in the reactive astrocytes at 48 h post-surgery. They hypothesized that reactive astrocytes induced by ischemia and stab wounding model go through a similar delayed up-regulation of PAC1 receptor in the process of neuroprotection [69]. PACAP38 uptake by the brain following CCI treatment was observed by Rhea et al. [70] using radioactively labeled PACAP38. Transport of radioactively labeled PACAP38 was measured 2 hours, 24 hours, 72 hours, 1 week, 2 weeks and 4 weeks post-injury. The results suggested that changes in PACAP38 transport following TBI are not as marked as after other CNS injuries, like ischemic injury or lesion of the spinal cord. There was a double- to 3-fold increase in transport rate three days after CCI compared to the initial measurements. The cerebellum had an approximately 2-fold greater transport rate for PACAP38 compared to the cortex and the entire brain. These results show that PACAP38 transport is temporally altered following CCI-treatment and PACAP38 uptake is greater in the cerebellum than in the cerebral cortex [70].

These results are in accordance with several other observations showing upregulated PACAP levels after traumatic nerve injuries [20]. In a most recent study, a significant upregulation of PACAP and PAC1-R was found in the retina after optic nerve crush [71]. All these data indicate the potential endogenous protective effects of PACAP, as elevated levels could provide increased endogenous protection. This possibility is further highlighted by the observations in PACAP gene deficient mice, where lack of the endogenous peptide increases the vulnerability in different insults [72]. It is not known whether PACAP knockout mice are also more sensitive to TBI, although in a contusion spinal cord injury model Tsuchikawa et al. [73] proved the neuroprotective effect of endogenous PACAP. PACAP and PAC1 receptor mRNA and immunoreactivity increased in the spinal cord after injury. The degree of spinal cord injury in PACAP knockout mice was enhanced compared with that seen in wild-type mice. This was also reflected in functional impairment: wild-type mice gradually recovered locomotor function after day three, while knockout mice performed much worse in motor function throughout the two-week observation period. These results suggest that PACAP present endogenously is able to suppress the loss of motor function by inhibiting neuronal cell death after injury of the spinal cord [73]. The age-related physiological or pathology-associated decline in endogenous PACAP levels could also contribute to the increased vulnerability of these individuals to brain trauma [17].

## 5. PACAP Levels in the Brain, CSF and Serum in TBI Patients

Human brain tissues were investigated from medico-legal autopsy cases by van Landeghem et al. [58] in human TBI. The victims were classified into three groups dependent on their survival time (under 24 hours, between 24 h and 7 days, and between 7 and 99 days post-injury). During the immunohistochemical analysis PACAP27 and PACAP38 expressing cells were counted. Neuronal and glial cells exhibited a strong cytoplasmic immunopositivity for PACAP27 or PACAP38 in all cortical layers of the examined lobes in controls. The analysis demonstrated an immediate and prolonged decrease of cellular PACAP27 and PACAP38 immunoreactivity in the contusion regions and a significantly increased PACAP27 and PACAP38 immunoreactivity in the pericontusional cortex at all survival times examined. A significant difference in the reaction type and/or extent between PACAP27 and PACAP38 was not seen. The prolonged post-traumatic increase of PACAP27 and PACAP38 immunoreactivity in reactive astrocytes may be interpreted as part of a complex endogenous neuroprotective reaction of astrocytes following TBI [58]. Presence of PACAP and its level in the plasma and in the CSF in severe TBI patients and non-head injured human controls were compared by Bukovics et al. [59]. Human blood and CSF samples were taken from patients every day. Samples were collected from individuals who suffered severe (Glasgow Coma Scale ≤ 8 on admission) TBI. The relationship between the time course of plasma PACAP and CSF levels and the final trauma outcome were investigated. Elevated plasma and CSF levels were measured in severe head injury patients compared to the controls in both CSF and blood plasma. The authors observed that there was a tendency of higher (nearly double) plasma levels in patients who died within the first week after TBI. The values were almost doubled during the entire period compared to the CSF levels of the same patients. Values were also analyzed in relation to the plasma and CSF levels of surviving patients and it was found that these levels were significantly lower compared to the high plasma levels of patients who died during the first week after TBI [59]. Concerning the source of the extra amount of PACAP in the aforementioned biological fluids of these patients compared to the normal controls they hypothesized that the trauma related endogenous overproduction, the damage of the blood-brain barrier, the secondary CNS injuries, the systemic inflammatory response syndrome and the elevated ceruloplasmin (PACAP-binding protein in the serum) concentration together can be responsible for the observed prolonged elevation. The authors concluded that PACAP appears less effective than the currently known and potential sets of protein biomarkers, but their results still call for further precisely focused investigations regarding this use of PACAP38 as a biomarker in severe TBI [59].

## 6. Concluding Remarks

**Figure 1:**
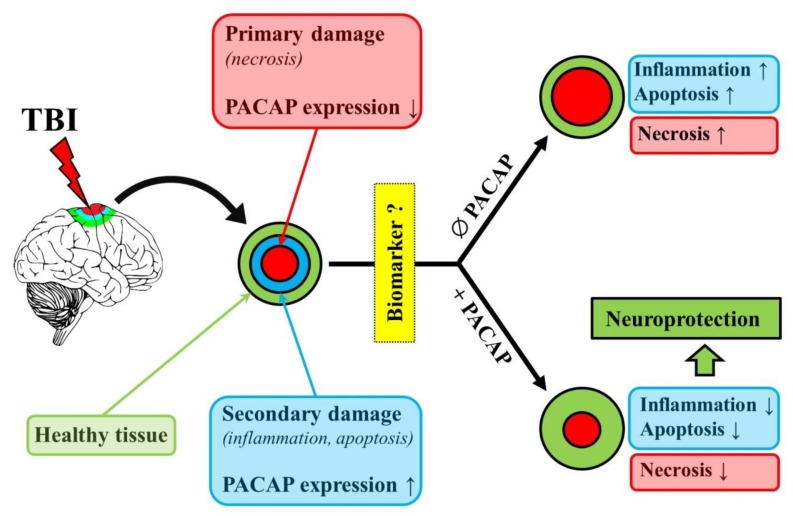
The neuroprotective and biomarker potential of pituitary adenylate cyclase activating polypeptide (PACAP). Red boxes and circles indicate primary damage after traumatic brain injury (TBI), blue boxes and circles show secondary damage, while green boxes and circles indicate healthy tissue or the process (neuroprotection) leading towards regenerated tissue.

In the acute management for TBI patients the standard medical and surgical interventions play a significant role. There is a lack of real effective treatment for recovery, this calls for research of TBI to be shifted into the area of prevention, treatment of secondary brain injury and neurorehabilitation. The importance of PACAP in the neuronal regeneration lies in the upregulation of endogenous PACAP and its receptors and the protective effect of exogenous PACAP after different central nervous system injury. The animal models not only can help us understand the pathophysiology of TBI, but allow us to develop interventions for preventing secondary injury, enhancing brain repair and improving recovery after TBI [74]. The results of the aforementioned animal experiments indicate that PACAP may also be a promising therapeutic agent in case of TBI due to its anti-inflammatory, anti-apoptotic and anti-oxidant effects (Figure 1). 

Regarding the biomarker value of PACAP, an increasing amount of evidence suggests the high translational potential of PACAP as a diagnostic and/or prognostic biomarker, especially in subprocesses like extent of the blood-brain barrier disruption, or the state of the systemic inflammatory response syndrome. The expanding number of publications in the last few years dealing with the role of PACAP as a novel biomarker showing that it is a rapidly developing, hot and promising topic. We believe that future studies will contribute to a better understanding of the possible role(s) of PACAP in human TBI and could serve as a good source for multi-center clinical trials which involve this topic.
